# Dorsal Raphe Serotonergic Neurons‐Ventral Tegmental Area Neural Pathway Promotes Wake From Sleep

**DOI:** 10.1111/cns.70141

**Published:** 2024-11-26

**Authors:** Yuhao Wang, Dan Wang, Xinxin Zhang, Huiming Li, Sa Wang, Yuting He, Guangchao Zhao, Hailong Dong, Jiannan Li

**Affiliations:** ^1^ Department of Anesthesiology and Perioperative Medicine Xijing Hospital, Fourth Military Medical University Xi'an Shaanxi China; ^2^ Shaanxi University of Chinese Medicine Xianyang Shaanxi China

**Keywords:** dorsal raphe nucleus, neuronal firing, serotonin, sleep, ventral tegmental area, wake

## Abstract

**Background:**

Dorsal raphe nucleus (DRN) serotonergic neurons projecting to the ventral tegmental area (VTA) neural circuit participate in regulating wake‐related behaviors; however, the effect and mechanism of which in regulating sleep–wake are poorly understood.

**Methods:**

Fiber photometry was used to study DRN serotonergic afferent activity changes in the VTA during sleep–wake processes. Optogenetics and chemogenetics were took advantage to study the effects of DRN serotonergic afferents modulating VTA during sleep–wake. In vivo electrophysiology was employed to investigate how VTA neuronal firings were influenced by upregulation of DRN serotonergic afferents during sleep–wake.

**Results:**

We found that DRN serotonergic afferent activity in the VTA was higher during wake than during NREM and REM sleep. Chemogenetic activation of VTA‐projecting DRN serotonergic neurons increased wake, and optogenetic activation of DRN serotonergic terminals in the VTA induced wake during NREM and REM sleep. Furthermore, we found that optogenetic activation of DRN serotonergic terminals in the VTA increased glutamatergic neuronal firing, decreased dopaminergic neuronal firing, but not influenced GABAergic neuronal firing during NREM sleep.

**Conclusion:**

Our findings provide evidence in understanding the role of DRN serotonergic neurons‐VTA neural pathway in regulating sleep–wake, in which dynamic VTA dopaminergic, glutamatergic, and GABAergic neuronal firing changes responded to the wake promoting effect of DRN serotonergic afferents.

## Graphical abstract

DRN serotonergic afferent activity in the VTA are wake active. Upregulation of DRN serotonergic terminals in the VTA induced wake from sleep. Dynamic VTA dopaminergic, glutamatergic, and GABAergic neuronal firing changes respond to the wake‐promoting effect of DRN serotonergic afferents.

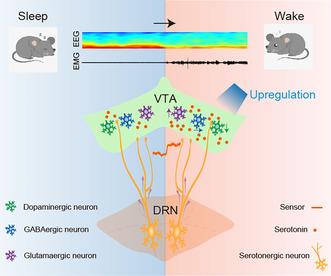

## 1 Introduction

Dorsal raphe nucleus (DRN) serotonergic neurons involve in a wide range of neurophysiological regulation, including sleep and wake [1, 2, 3]. However, the role of DRN serotonergic neurons in sleep is controversial. Previous studies found that optogenetic activation of DRN serotonergic neurons promoted arousal from sleep [4, 5] whereas tonic activation of which increased non‐rapid eye movement (NREM) sleep in zebrafish and mice [6]. Meanwhile, DRN serotonergic neurons were considered to modulate orexin neurons to maintain wakefulness and rapid eye movement (REM) sleep [7]. These studies indicate that the effect and mechanism of DRN serotonergic neurons and relevant neural circuits in regulating sleep–wake are intricate.

DRN serotonergic neurons project to multiply brain regions including the ventral tegmental area (VTA) [8, 9]. VTA mainly contains dopaminergic, glutamatergic, and γ‐aminobutyric acid (GABA)‐ergic neurons is considered to be a reward brain centre [10, 11, 12]. Previous studies indicated that VTA dopaminergic and glutamatergic neurons involve in promoting arousal while GABAergic neurons participate in promoting sleep [13, 14]. Meanwhile, DRN serotonergic neurons could regulate reward and feeding behaviors via modulating the VTA [15, 16, 17]. However, whether and how DRN serotonergic neurons modulate VTA neurons to regulate sleep–wake remain poorly understood.

Here, we took advantage of fiber photometry, chemogenetics, optogenetics, and in vivo electrophysiology to investigate the role of DRN serotonergic neurons modulating VTA neurons in sleep–wake. First, we observed DRN serotonergic afferent activity changes in the VTA during sleep–wake process. Subsequently, we observed the effects of chemogenetic and optogenetic modulating DRN serotonergic neurons‐VTA neural pathway during sleep–wake. Finally, we investigated how the VTA neuronal firings were influenced by activation of DRN serotonergic terminals in the VTA during sleep.

## 2 Methods

### 2.1 Animals

C57BL/6J wild‐type mice were provided by Vital River Laboratory Animal Technology. Sert‐Cre mice were generously provided by Prof. Minmin Luo (National Institute of Biological Sciences, Beijing, China) and were bred in our lab. Mice were housed under specific‐pathogen‐free conditions with constant temperature (21°C–23°C) and humidity (38%–42%) on a light‐controlled schedule (lights on at 7:00 AM and lights off at 7:00 PM) with free access to food and water. All experiments were performed during the light‐on period. A total of 63 mice aged 6–8 weeks were used for this study, valid data were obtained from 36 Sert‐Cre mice (19 male and 17 female) and 18 C57BL/6J wild‐type mice (10 male and 8 female). Male and female mice were experimented for sleep recording, respectively, to avoid influencing with each other. Data from two mice with failed expressing the virus and six mice with untargeted placement of fibers were excluded. The experimental protocol was approved by the Ethics Committee for Animal Experimentation and was conducted in accordance with the Guidelines for Animal Experimentation of the Fourth Military Medical University.

### 2.2 Surgical Procedure and Virus Injection

Mice were fixed in a stereotaxic frame (RWD, Shenzhen, China) under 1.4% isoflurane (Baxter Healthcare Corporation, USA) anesthesia with pure oxygen (flow rate 0.5 L/min), and body temperature of mice was maintained by using a heating pad. The eyes were protected by erythromycin ophthalmic ointment. Following shaving off the hair and disinfecting the incision site using iodine, 3% hydrogen peroxide was used to remove the fascia from the skull surface. The bregma and lambda points were used to adjust the mouse head to the horizontal position.

The virus was provided by BrainVTA Technology Co. Ltd. (Wuhan, China). The microinjection flow rate of virus was set at 50 nL/min.

For anterograde tracing, 200 nL Cre‐dependent adeno‐associated viruses expressing mCherry (*AAV*_*2/9*_ *‐EF1α‐DIO‐mCherry*) was microinjected into the DRN (anterior–posterior [AP]: −4.55 mm; medial‐lateral [ML]: 0 mm; dorsal‐ventral [DV]: −2.55 mm) in Sert‐Cre mice. For retrograde tracing, 200 nL Cre‐dependent adeno‐associated viruses expressing mCherry (*AAV*_*2/retro*_ *‐EF1α‐DIO‐EGFP*) was bilaterally microinjected into the VTA (AP: −3.3 mm; ML: ±0.4 mm; DV: −4.0 mm) in Sert‐Cre mice. Totally, two male and two female Sert‐Cre mice were used for anterograde and retrograde without virus expression failure.

For fiber photometry, 200 nL Cre‐dependent adeno‐associated viruses expressing GCaMP6s (*AAV*_*2/9*_ *‐Ef1a‐DIO‐GCaMP6s‐WPRE‐pA*) or 5‐HT3.0 (*AAV*_*2/9*_ *‐Ef1a‐DIO‐5‐HT3.0*) were microinjected into the DRN in Sert‐Cre mice. Subsequently, an optical fiber (diameter: 200 μm, Inper, Hangzhou, China) was inserted into the ipsilateral VTA. Eventually, a TL11M2‐F20‐EET device (Data Science International) was implanted in the subcutaneous cavity on the back of mice, four wires of which were subcutaneously led to the mouse's neck by a guiding cannula. A pair of wires was imbedded into the bilateral parietal skulls (AP, +0.2 mm, ML, +1.5 mm, DV −0.1 mm; AP −1.7 mm, ML −0.2 mm, DV −0.1 mm) to record EEG. The other pair of wires was implanted in the neck muscles to monitor the electromyography (EMG). Then, the optical fiber, wires, and skull were fixed with dental cement. Totally six male and eight female Sert‐Cre mice were used for fiber photometry in which two female mouse was found untargeted placement of fibers.

For optogenetic modulations, 200 nL Cre‐dependent adeno‐associated viruses expressing channelrhodopsin 2 (ChR2) (*AAV*_*2/9*_ *‐Ef1a‐DIO‐hChR2‐mCherry*) or control virus (*AAV*_*2/9*_ *‐Ef1a‐DIO‐mCherry*, mCherry) were microinjected into the DRN in Sert‐Cre mice. An optical fiber was inserted into the VTA, subsequently, EEG and EMG were implanted. Then, the optical fiber, wires, and skull were fixed with dental cement. Totally, seven male and seven female Sert‐Cre mice were used for optogenetics in which two female mice were found untargeted placement of fibers. And, two female mice were used for in vitro electrophysiology.

For chemogenetic modulations, 200 nL Cre‐dependent adeno‐associated viruses expressing hM3Dq (*AAV*_*2/retro*_ *‐Ef1a‐DIO‐hM3Dq‐mCherry*), hM4Di (*AAV*_*2/retro*_ *‐Ef1a‐DIO‐hM4Di‐mCherry*), or control virus (*AAV*_*2/retro*_ *‐Ef1a‐DIO‐mCherry*, mCherry) were microinjected into the VTA, and AAV_2/9_‐Tph2‐Cre were microinjected into DRN in C57BL/6J mice. Then EEG and EMG were implanted and were fixed with dental cement. Totally 11 male and 10 female C57BL/6J mice were used for chemogenetics in which one male mouse and two female mice were found virus expression failure.

For in vivo electrophysiology, 200 nL Cre‐dependent excitatory optogenetic virus (*AAV*_*2/9*_ *‐Ef1a‐DIO‐hChR2‐EGFP*) was injected into the DRN and 200 nL inhibitory optogenetic virus with tyrosine hydroxylase promoter (*AAV*_*2/9*_ *‐TH‐NpHR‐mCherry*) was injected into the VTA in Sert‐Cre mice, simultaneously. Opto‐tetrode with 16 channels (Kedou, Suzhou, China) was implanted into the VTA two weeks later to record spike, local field potential, EEG, and EMG signals. Totally, five male and three female Sert‐Cre mice were used for in vivo electrophysiology in which one male mouse and one female mouse virus were found untargeted placement of fibers.

At termination of surgery, 1% lidocaine (0.5 mL) was subcutaneously injected. Three weeks were allowed for mice recovery and virus expression.

### 2.3 Immunofluorescence Staining

Mice were deeply anesthetized with isoflurane and cardially perfused with 4% paraformaldehyde (PFA) followed by 0.9% cold saline. Brains were post‐fixed for 2 h in 4% PFA at 4°C temperature and then dehydrated by 30% sucrose in phosphate‐buffered saline (PBS) serially. Brains (containing the DRN and the VTA) were coronally sectioned into 40 μm slices by using a cryostat microtome (Leica, CM1900, Germany). The brain slices were washed in PBS and then blocked with 5% normal donkey serum (NDS) in PBS with 0.3% Triton X‐100 (PBST) for 2 h at room temperature. Primary antibodies including rabbit anti‐tryptophan 5‐hydroxylase 2 (1:200, Synaptic Systems, USA) were incubated in PBST containing 2.5% NDS at 4°C for 24 h. Secondary antibodies including donkey anti‐rabbit Alexa 488 (1:500, Jackson ImmunoResearch) or donkey anti‐mouse Alexa 647 (1:500, Jackson ImmunoResearch) were incubated in PBST containing 2.5% NDS at 24°C for 2 h. Subsequently, slices were imaged by a laser confocal fluorescence microscope (FV1200, Olympus, Japan). The mice with untargeted placement of fibers were excluded from experiments. Fluorescence intensities of each region of whole brain were analyzed.

Images were background subtracted and then binarized based on a pixel‐intensity threshold that was held constant for all samples analyzed. Regions of interest were then drawn manually and were defined based on background signal. Axon density is reported as ((red pixels in region of interest)/(total pixels in region of interest)) × 100.

### 2.4 Fiber Photometry

Optical fiber was attached to the fluorescence photometer (Thinker Tech, Nanjing, China). A 488‐nm LED light beam with intensity of 30–40 μW was used to evoke and record the serotonin sensor fluorescence signals during sleep–wake and isoflurane anesthesia‐arousal process. The signal data were extracted and analyzed by MATLAB R2018b (MathWorks, USA). The Δ*F*/*F* was calculated as (*F* – *F*_0_)/*F*_0_ × 100, where *F*_0_ was the baseline fluorescence signal and *F* was the real‐time fluorescence signal. Δ*F*/*F* values were expressed as percentages and presented in average plots with a shaded area indicating the standard error of the mean (SEM). Fluorescence signals of different states in sleep–wake process were averaged and compared. Fluorescence signals that 30 s pre‐ and post‐state alternations in sleep–wake switches were analyzed.

### 2.5 Optogenetics and Chemogenetics

Optogenetic stimulation was performed with a laser (473 or 594 nm, Thinker Tech, Nanjing, China). The laser intensity at the fiber tip was measured using an optical power meter and was maintained between 10 and 15 mW. On the basis of previous studies [18] and the optimal stimulation frequency in in vitro electrophysiological tests, optogenetic activation was performed using a 473 nm laser at 20 Hz with a duration of 20 ms. Optogenetic stimulation was performed for a duration of 30 s during NREM/REM sleep.

For chemogenetic modulation, clozapine‐N‐oxide (CNO, 1 mg/kg, Cayman Chemical) was intraperitoneally injected 30 min before sleep–wake recording.

### 2.6 EEG/EMG Recording and Analysis

EEG and EMG signals were recorded at a digital sampling rate of 1000 Hz by using TL11M2‐F20‐EET telemetry devices and dataquest ART (version 4.33) or in vivo electrophysiology (BlackRock system, USA). Raw EEG data were bandpass filtered at 0.3–50 Hz with 50 Hz notched and EMG data were bandpass filtered at 10–200 Hz for subsequent analysis. EEG data during laser stimulation process were additionally notched by 20 and 40 Hz to remove laser frequency interference. NREM sleep, REM sleep, and wake states were automatically classified using a sleep analysis software NeuroScore (Version 3.3.1) and artificially adjusted.

To calculate the power spectrum, EEG signal was classified into five frequency bands as follows: delta (δ: 0.3–4 Hz), theta (θ: 4–10 Hz), alpha (α: 10–15 Hz), beta (β: 15–25 Hz), and gamma (γ: 25–50 Hz). The relative power of each frequency band was calculated as the percentage of the total power of 0.3–50 Hz.

### 2.7 In Vivo Electrophysiology

Raw in vivo electrophysiological data were recorded with a band‐pass filter (250–5000 Hz) to obtain neuronal spikes and a low‐pass filter (250 Hz) to obtain the local field potential (LFP), EEG, and EMG values. Single units were sorted according to a threshold and shape detector using principal component analysis with offline sorter software (KlustaKwik, USA). The first two principal components of each spike on the two‐dimensional plot of the detected spike events were extracted. Waveforms with similar principal components were clustered using a K‐means sorting method. The isolated cluster was considered as a single unit recorded from the same neuron. Spikes with inter‐spike intervals < 2 ms were discarded. Cross‐correlation histograms were used to eliminate cross‐channel artifacts.

To identify dopaminergic neurons in the VTA, 1 Hz 594 nm laser pulse trains for 10 s were applied at the wake states during sleep–wake. Clusters that were inhibited during laser application were identified as dopaminergic neuronal firings. GABAergic neuronal firings in the VTA were distinguished from dopaminergic neuronal firings based on their relatively high rapid‐firing, non‐bursting activity, and short‐duration action potentials (< 0.5 ms) [19]. There was another group of neuronal firing waveforms that were different from those of dopaminergic and GABAergic neuronal firing, and we designated them as glutamatergic neurons. NeuroExplorer software (version 5.0) was used to analyze the neuronal firing, EEG, and LFP changes.

### 2.8 Statistical Analysis

GraphPad Prism 8.0.1 (GraphPad Software Inc., CA, USA) was used for statistical analysis. Latency to wake and time spend in different sleep states data were expressed as the Mean [Standard Deviation] and analyzed with a two‐tailed unpaired Student *t* test. Fiber photometry signal, EEG, and neuronal firing data were expressed as the Mean [Standard Error of Mean] and analyzed with a two‐tailed unpaired/paired Student *t* test or repeated measurement/ordinary one‐way analysis of variance (ANOVA) with a Tukey test. *p < 0.05, **p < 0.01, ***p < 0.001was considered to be statistically significant in all cases.

## 3 Results

### 3.1 DRN Serotonergic Afferents in the VTA Are Wake Active

**FIGURE 1:**
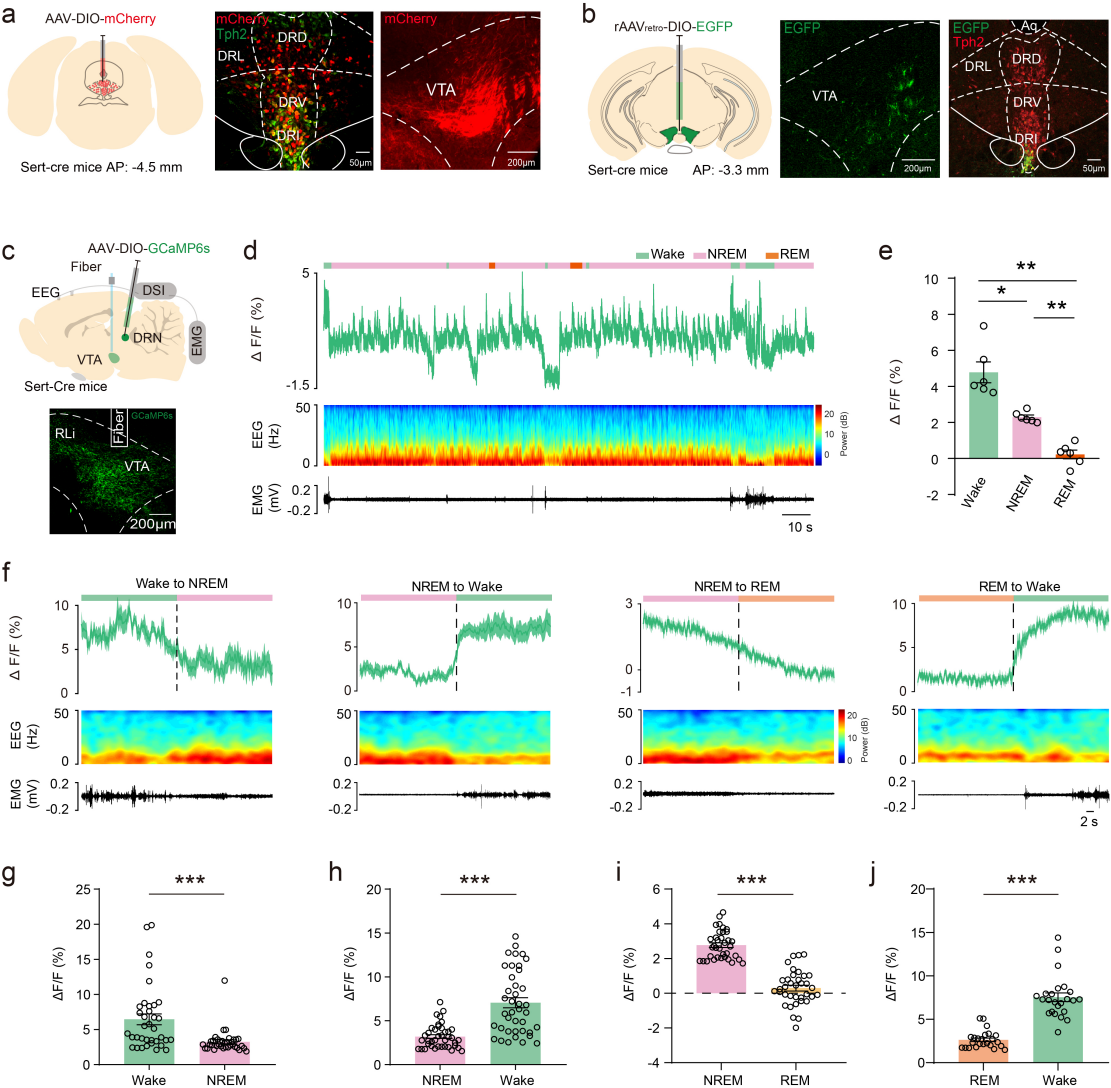
DRN serotonergic terminal activity changes in the VTA during sleep–wake. (a) Schematic diagram of anterograde tracing virus injection (Left), image of virus expression overlaps with serotonergic immunofluorescence staining in the DRN (Middle), and image of serotonergic terminal expressing in the VTA (Right). (b) Schematic diagram of retrograde tracing virus injection (Left), image of retrograde tracing virus expressing in the VTA(Middle), and image of retrograde tracing virus expression overlaps with serotonergic immunofluorescence staining in the DRN (Right). (c) Schematic of virus injection, fiber, and DSI implantation and image of GCaMP6s expression and optical fiber cannula in the VTA. (d) Representative raw VTA GCaMP6s fluorescence intensity trace and relevant EEG power spectra/EMG traces during sleep–wake. (e) Quantification of VTA GCaMP6s fluorescence intensity during wake, NREM, and REM. (f) Representative raw VTA GCaMP6s fluorescence intensity trace and relevant EEG power spectra/EMG traces in states switches during sleep–wake. (g) Quantification of VTA GCaMP6s fluorescence intensity in wake switch to NREM sleep. (h) Quantification of VTA GCaMP6s fluorescence intensity in NREM sleep switch to wake. (i) Quantification of VTA GCaMP6s fluorescence intensity in NREM sleep switch to REM sleep. (j) Quantification of VTA GCaMP6s fluorescence intensity in REM sleep switch to wake. DR, dorsal raphe; EEG, electroencephalogram; EMG, electromyography; VTA, ventral tegmental area. *p < 0.05, **p < 0.01, ***p < 0.001.

To confirm the connection between DRN serotonergic neurons and VTA, we injected anterograde tracing viruses (*AAV*_*2/9*_ *‐EF1α‐DIO‐mCherry*, 200 nL) into the DRN in Sert‐Cre mice. We found that DRN serotonergic neurons projected to numerous brain regions including the medial prefrontal cortex, nucleus accumbens, basal forebrain, lateral habenula, lateral hypothalamus, and the VTA (Figure 1a and Figure S1). By injecting retrograde tracing viruses (*AAV*_*2/retro*_ *‐EF1α‐DIO‐EGFP*, 200 nL) into the VTA in Sert‐Cre mice, we further found that VTA projecting serotonergic neurons were mainly distributed in the interfascicular part of DRN (Figure 1b).

Then, calcium signal viruses (*AAV*_*2/9*_ *‐EF1α‐DIO‐GCaMP6s*, 200 nL) were injected into the DRN, fiber was implanted above the VTA simultaneously in Sert‐Cre mice, and fiber photometry was utilized to monitor DRN serotonergic terminal activity changes in the VTA during sleep–wake (Figure 1c–e). DSI was implanted to record electroencephalogram (EEG) and electromyography (EMG). We found that DRN serotonergic terminal activity in the VTA was higher during wake than during NREM and REM sleep (Wake: 4.779% ± 0.5784% vs. NREM: 2.292% ± 0.1178% vs. REM: 0.2266% ± 0.2411%, *n* = 6, *F* = 38.34, *p* < 0.001; Figure 1d,e). DRN serotonergic terminal activity in the VTA decreased from wake switch to NREM sleep (Wake: 6.458% ± 0.7747% vs. NREM: 3.255% ± 0.2755%; *n* = 36 trails from 6 mice, *p* < 0.001; Figure 1f,g) and from NREM switch to REM sleep (NREM: 2.767% ± 0.1352% vs. REM: 0.2967% ± 0.1654%; *n* = 36 trails from 6 mice, *p* < 0.001; Figure 1f,i). Conversely, DRN serotonergic terminal activity in the VTA increased from NREM sleep switch to wake (NREM: 3.194% ± 0.2123% vs. Wake: 7.052% ± 0.5747%; *n* = 40 trails from 6 mice, *p* < 0.001; Figure 1f,h) and from REM sleep switch to wake (REM: 0.2636% ± 0.2053% vs. Wake: 7.538% ± 0.4978%; *n* = 24 trails from 6 mice, *p* < 0.001; Figure 1f,j). Similar changes were also observed of serotonin sensor activity in the VTA during sleep–wake cycle (Figure S2). These findings demonstrate that DRN serotonergic afferents in the VTA are wake active during sleep–wake process.

### 3.2 Chemogenetic Activation of VTA Projecting DRN Serotonergic Neurons Increases Wake

**FIGURE 2:**
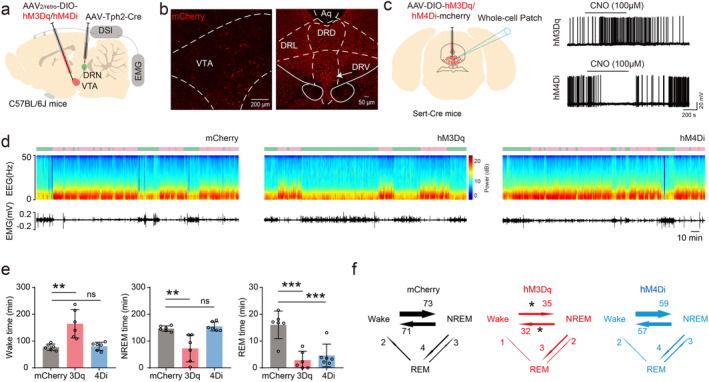
Effects of chemogenetic modulation of VTA‐projecting DRN serotonergic neurons during sleep–wake. (a) Schematic of chemogenetic virus injection and DSI implantation. (b) Image of retrograde chemogenetic virus expression in the VTA (Left) and DRN (Right). (c) Schematic of chemogenetic virus injection and whole cell patch (Left), effect of CNO on hM3Dq and hM4Di in vitro. (d) Representative EEG and EMG changes of chemogenetic modulation in sleep–wake cycle. (e) Quantification of time spent in different states in sleep–wake cycle under chemogenetic modulation. (f) Quantification of time of different states switches in sleep–wake cycle under chemogenetic modulation. 3Dq, hM3Dq; 4Di, hM4Di; CNO, clozapine‐N‐oxide; DR, dorsal raphe; VTA, ventral tegmental area.*p < 0.05, **p < 0.01, ***p < 0.001.

To investigate the long‐term effect of DRN serotonergic neurons‐VTA neural pathway during sleep–wake, chemogenetic viruses (*AAV*_*2/retro*_ *‐Ef1a‐DIO‐hM3Dq‐mCherry*, *AAV*_*2/retro*_ *‐Ef1a‐DIO‐hM4Di‐mCherry* or *AAV*_*2/retro*_ *‐Ef1a‐DIO‐mCherry*, 200 nL each) were microinjected into the VTA, separately, and viruses with serotonergic promoter (*AAV*_*2/9*_ *‐Tph2‐Cre*, 200 nL) were microinjected into the DRN in C57BL/6J mice (Figure 2a,b). Clozapine‐N‐oxide (CNO) (1 mg/kg) were intraperitoneally injected to modulate the activity of neurons infected by chemogenetic viruses (Figure 2c). We found that chemogenetic activation of VTA projecting DRN serotonergic neurons significantly increased the time spend in wake (hM3Dq: 164.5 ± 52.79 min vs. mCherry: 77.27 ± 11.62 min; *n* = 6, *p* < 0.001), decreased the time spend in NREM sleep (hM3Dq: 72.57 ± 50.38 min vs. mCherry: 146.7 ± 9.802 min; *n* = 6, *p* = 0.0024) and REM sleep (hM3Dq: 2.867 ± 3.354 min vs. mCherry: 16.07 ± 5.158 min; *n* = 6, *p* < 0.001) compared with control (Figure 2d,e). Chemogenetic inhibition of VTA projecting DRN serotonergic neurons did not influence the time spent in wake and NREM sleep, but decreased the time spent in REM sleep compared with control (hM4Di: 4.633 ± 4.233 min vs. mCherry: 16.07 ± 5.158 min; *n* = 6, *p* = 0.001; Figure 2d,e).

Moreover, chemogenetic activation of VTA projecting DRN serotonergic neurons significantly decreased the transitions between wake and NREM sleep (Wake to NREM, hM3Dq: 34.83 ± 18.98 vs. mCherry: 72.83 ± 9.802; *n* = 6, *p* = 0.0469; NREM to Wake, hM3Dq: 31.50 ± 18.51 vs. mCherry: 70.83 ± 24.31; *n* = 6, *p* = 0.0184; Figure 2d,f). These results indicate that chemogenetic activation of VTA‐projecting DRN serotonergic neurons enhances and stabilizes wake.

### 3.3 Optogenetic Activation of DRN Serotonergic Terminals in the VTA Induces Wake From Sleep

**FIGURE 3:**
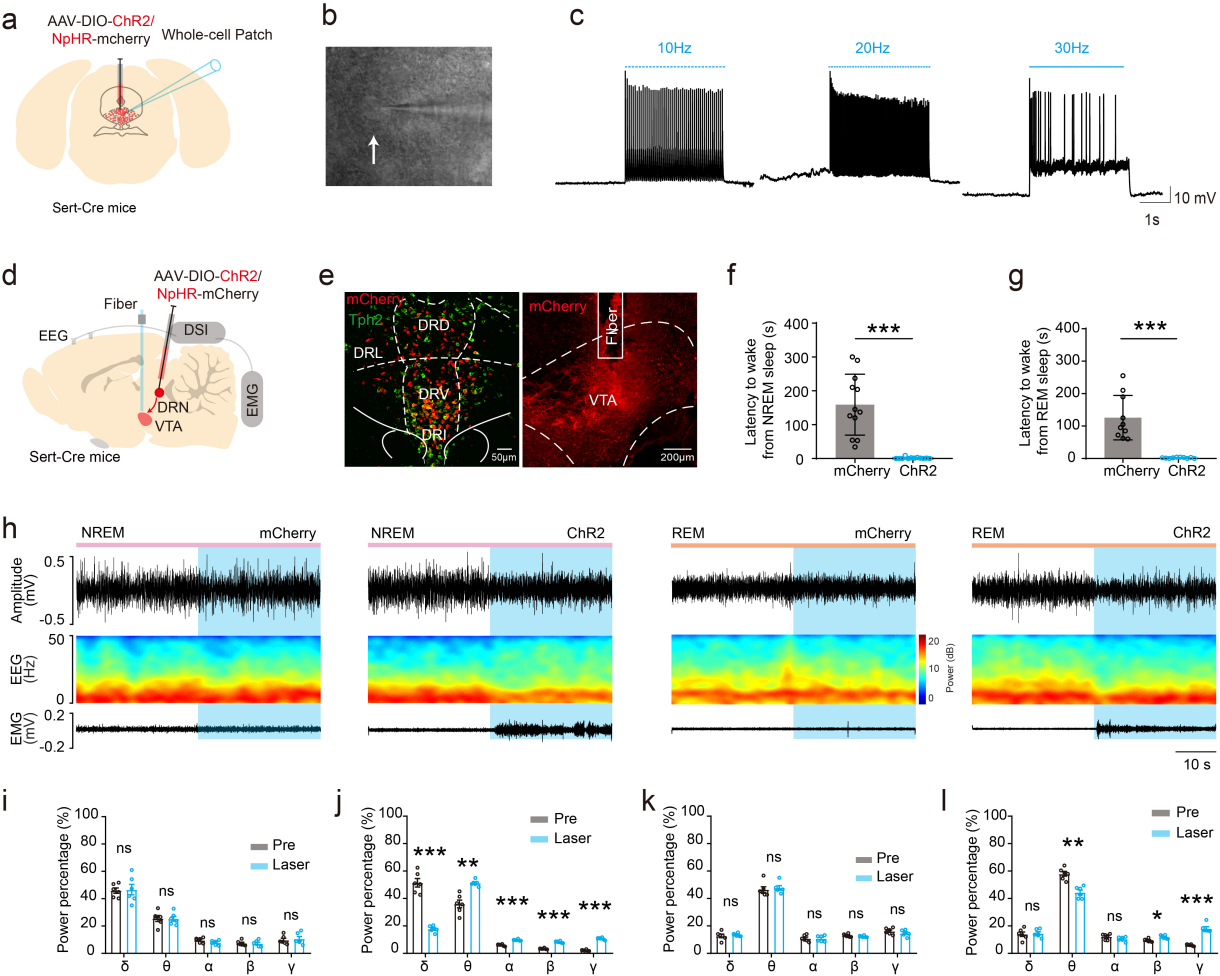
Effects of optogenetic activation of DRN serotonergic terminals in the VTA during sleep. (a) Schematic of optogenetic virus injection and whole‐cell patch. (b) Representative image of DRN serotonergic neurons and whole‐cell patch. (c) Verification of optogenetic stimulation parameters by 473 nm laser at different frequencies (blue bars represent 20 ms laser pulses). (d) Schematic of optogenetic virus injection, fiber, and DSI implantation. (e) Image of optogenetic virus expression overlaps with serotonergic immunofluorescence staining in the DRN (Left) and optical fiber cannula in the VTA (Right). (f) Quantification of latency from NREM sleep to wake after optogenetic activation of VTA serotonergic terminals compared with control group. (g) Quantification of latency from REM sleep to wake after optogenetic stimulation compared with control group. (h) Representative EEG and EMG changes induced by optogenetic stimulation during NREM and REM. (i) Quantification of EEG power changes by optogenetic activation DRN serotonergic terminals in the VTA during NREM sleep in control group. (j) Quantification of EEG power spectra changes after optogenetic stimulation during NREM sleep in experimental group. (k) Quantification of EEG power changes by optogenetic activation during REM sleep in control group. (l) Quantification of EEG power spectra changes after optogenetic stimulation during REM sleep in experimental group. DR, dorsal raphe; VTA, ventral tegmental area. *p < 0.05, **p < 0.01, ***p < 0.001.

To study the effect of DRN serotonergic neurons‐VTA neural pathway in sleep–wake states switch, optogenetic viruses (*AAV*_*2/9*_ *‐Ef1a‐DIO‐hChR2‐mCherry* or *AAV*_*2/9*_ *‐Ef1a‐DIO‐mCherry*, 200 nL each) were microinjected into the DRN separately and fiber was implanted above the VTA in Sert‐Cre mice. Laser stimulations (473 nm, 20 Hz, 10–15 mV) were performed to activate neuronal terminals infected by optogenetic viruses (Figure 3a–e). We found that optogenetic activation of DRN serotonergic terminals in the VTA during NREM sleep significantly decreased the latency to wake compared with control (ChR2: 1.417 ± 2.575 s vs. mCherry: 159.2 ± 89.91 s; *n* = 12 trails from 6 mice, *p* < 0.001; Figure 3f), at the same time, EEG changed to wake (Figure 3h–j). Similarly, optogenetic activation of DRN serotonergic terminals in the VTA during REM sleep significantly decreased the latency to wake compared with control (ChR2: 1.9 ± 1.595 s vs. mCherry: 159.2 ± 68.51 s; *n* = 12 trails from 6 mice, *p* < 0.001; Figure 3g), at the same time, EEG changed to wake (Figure 3h,k,l). These results indicate that optogenetic activation of DRN serotonergic terminals in the VTA is sufficient to promote wake from sleep.

### 3.4 VTA Neuronal Firings Heterogeneously Change During Sleep–Wake

**FIGURE 4:**
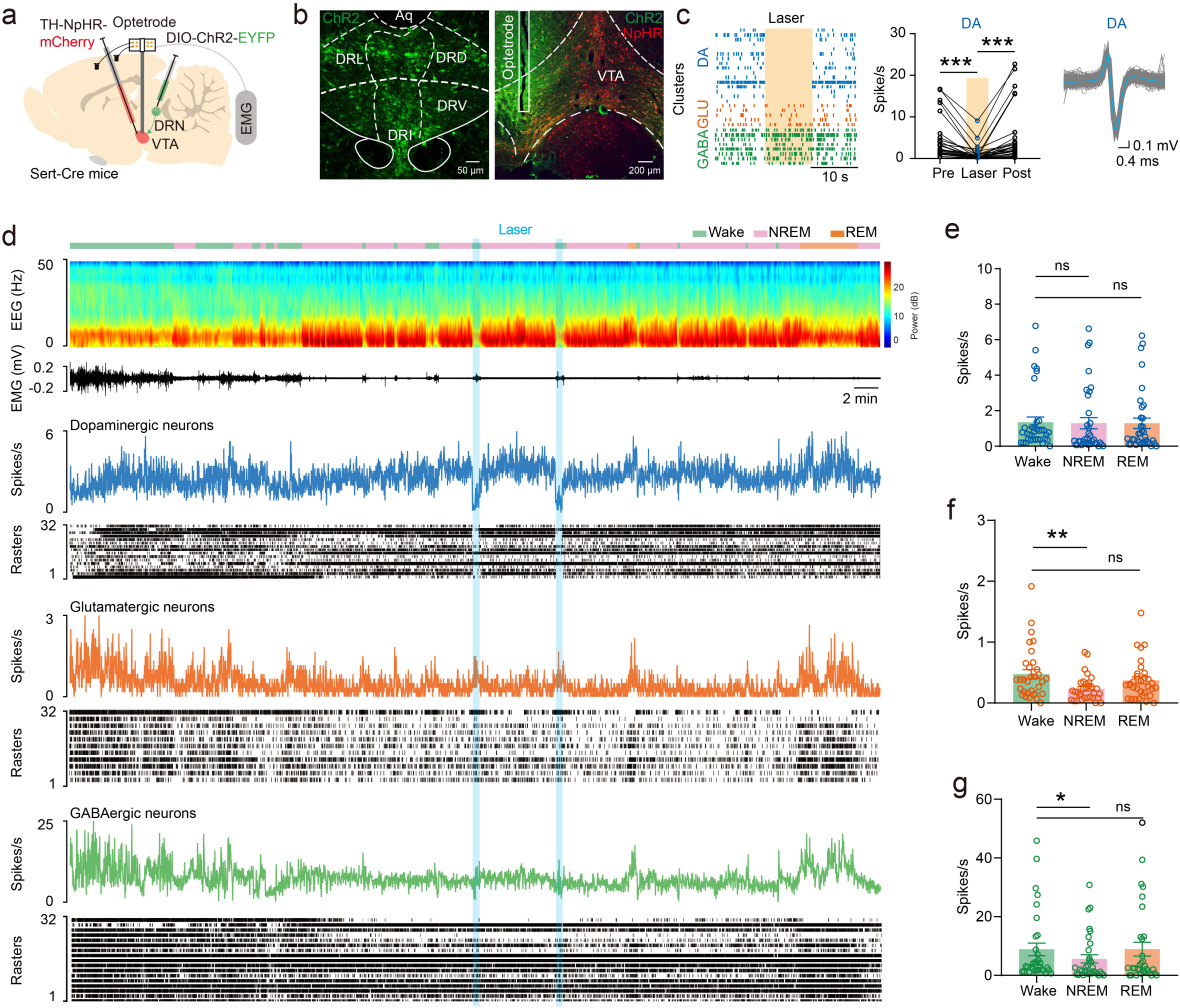
Changes of VTA neuronal firing during sleep–wake. (a) Schematic of optogenetic virus injection and opto‐tetrode implantation. (b) Image of optogenetic virus expression in the DRN (Left) and virus expression with opto‐tetrode cannula in the VTA (Right). (c) Raster diagram showing spikes that effectively suppressed by 594 nm laser pulse were identified as dopaminergic neuronal firing and glutamatergic and GABAergic neuronal firing were also identified based on firing characteristics (Left), quantification of VTA dopaminergic neuronal firing rate changes pre, during and post 594 nm laser pulse (Middle), and representative spike waveform of VTA dopaminergic neuronal firing (Right). (d) Representative VTA neuronal firing trances, rasters, and EEG/EMG changes during sleep–wake cycle within optogenetic stimulation. (e) Quantification of VTA dopaminergic neuronal firings during different states during sleep–wake. (f) Quantification of VTA glutamatergic neuronal firings during different states during sleep–wake. (g) Quantification of VTA GABAergic neuronal firings during different states during sleep–wake. DR, dorsal raphe; VTA, ventral tegmental area. *p < 0.05, **p < 0.01, ***p < 0.001.

In order to further investigate how VTA neuronal firing changed during sleep–wake and responded to the wake promoting effect of DRN serotonergic neurons, we took advantage of double virus system and in vivo electrophysiology. Cre‐dependent excitatory optogenetic viruses (*AAV*_*2/9*_ *‐Ef1a‐DIO‐hChR2‐EGFP*, 200 nL) were injected into the DRN and inhibitory optogenetic viruses with tyrosine hydroxylase promoter (*AAV*_*2/9*_ *‐TH‐NpHR‐mCherry*, 200 nL) were injected into the VTA in Sert‐Cre mice simultaneously, then opto‐tetrode was implanted into the VTA two weeks later to record spike, local field potential (LFP), EEG, and EMG signals (Figure 4a,b).

By the sensitivity to optogenetic inhibition (Pre: 4.231 ± 0.8327 Spikes/s vs. Laser: 0.9750 ± 0.3234 Spikes/s vs. Post: 4.953 ± 1.107 Spikes/s, *n* = 35, *F* = 20.40, *p* < 0.001; Figure 4c), 35 single units were identified as VTA dopaminergic neuronal firings. And, according to waveform principal component analysis and electrophysiologic properties, 32 single units were identified as glutamatergic neuronal firings and another 32 single units were identified as GABAergic neuronal firings.

We found that the firing rate of VTA dopaminergic neurons did not significantly change during sleep–wake (Wake: 1.357 ± 0.2875 Spikes/s vs. NREM: 1.297 ± 0.3142 Spikes/s vs. REM: 1.293 ± 0.2959 Spikes/s, *n* = 35, *F* = 0.03956, *p* = 0.9559; Figure 4d,e), while the firing rate of glutamatergic neurons (Wake: 0.4740 ± 0.07523 Spikes/s vs. NREM: 0.2328 ± 0.03648 Spikes/s, *n* = 32, *p* < 0.001; Figure 4d,f) and GABAergic neurons (Wake: 8.860 ± 2.112 Spikes/s vs. NREM: 5.641 ± 1.398 Spikes/s, *p* = 0.0363; Figure 4d,g) were higher in wake and REM sleep than in NREM sleep. These findings suggest that VTA neuronal firing changes during sleep–wake are heterogeneous.

### 3.5 Optogenetic Activation of DRN Serotonergic Terminals During Sleep Induces VTA Neuronal Firing Dynamic Change

**FIGURE 5:**
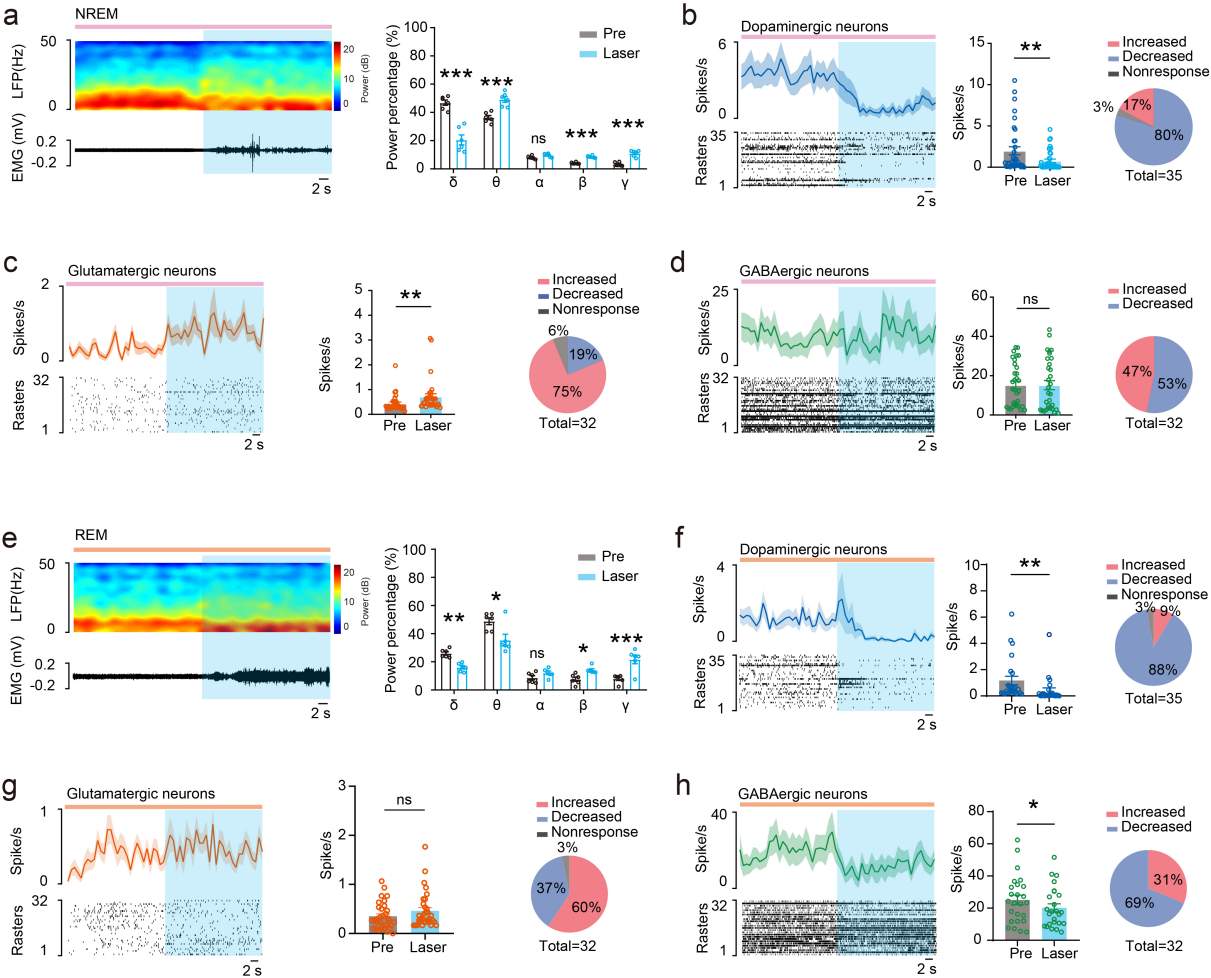
VTA neuronal firing changes induced by optogenetic activation of DRN serotonergic terminals during NREM sleep and REM sleep. (a) Representative VTA LFP/EMG changes (Left) and quantification of VTA LFP power changes by optogenetic stimulation during NREM sleep (Right). (b) Average firing (Left), quantification (Middle), and percentage (Right) changes of VTA dopaminergic neuronal firing by optogenetic stimulation during NREM sleep. (c) Average firing (Left), quantification (middle), and percentage (Right) changes of VTA glutamatergic neuronal firing by optogenetic stimulation during NREM sleep. (d) Average firing (Left), quantification (Middle), and percentage (Right) changes of VTA GABAergic neuronal firing by optogenetic stimulation during NREM sleep. (e) Representative VTA LFP/EMG changes (Left) and quantification of VTA LFP power changes by optogenetic stimulation during REM sleep (Right). (f) Average firing (Left), quantification (Middle), and percentage (Right) changes of VTA dopaminergic neuronal firing by optogenetic stimulation during REM sleep. (g) Average firing (Left), quantification (Middle), and percentage (Right) changes of VTA glutamatergic neuronal firing by optogenetic stimulation during REM sleep. (h) Average firing (Left), quantification (Middle), and percentage (Right) changes of VTA GABAergic neuronal firing by optogenetic stimulation during REM sleep. DR, dorsal raphe; VTA, ventral tegmental area. *p < 0.05, **p < 0.01, ***p < 0.001.

To investigate how DRN serotonergic neurons influenced neuronal firings in the VTA during promoting wake, laser stimulations (473 nm, 20 Hz, 10–15 mV) were performed during NREM and REM sleep. We found that optogenetic activation of DRN serotonergic terminals in the VTA during NREM sleep decreased the power percentage of δ band (from 46.82% ± 1.882% to 20.50% ± 3.485%; *n* = 6, *p* < 0.001) and increased the LFP power percentage of θ band (from 36.29% ± 1.471% to 49.18% ± 1.948%; *n* = 6, *p* < 0.001), β band (from 4.061% ± 0.2522% to 8.581% ± 0.3288%; *n* = 6, *p* < 0.001), and γ band (from 3.277% ± 0.5391% to 11.00% ± 0.8778%; *n* = 6, *p* < 0.001) of the VTA LFP, and effectively switched NREM sleep to wake (Figure 5a). Meanwhile, we found that optogenetic activation of DRN serotonergic terminals in the VTA decreased dopaminergic neuronal firing (from 1.991 ± 0.4893 Spikes/s to 0.7771 ± 0.2026 Spikes/s; *n* = 35, *p* = 0.0029; Figure 5b), increased glutamatergic neuronal firing (from 0.4385 ± 0.05996 Spikes/s to 0.7177 ± 0.1185 Spikes/s; *n* = 32, *p* = 0.0026; Figure 5c), but did not influence GABAergic neuronal firing (from 15.30 ± 1.938 Spikes/s to 15.19 ± 2.341 Spikes/s; *n* = 32, *p* = 0.9382; Figure 5d).

Furthermore, we found that optogenetic activation of DRN serotonergic terminals in the VTA during REM sleep decreased the power percentage of δ band (from 26.06% ± 1.186% to 16.11% ± 1.209%; *n* = 6, *p* = 0.0027) and θ band (from 48.91% ± 2.419% to 35.45% ± 4.158%; *n* = 6, *p* = 0.0232), and increased β band (from 7.628% ± 1.459% to 14.04% ± 0.9623%; *n* = 6, *p* = 0.0202), and γ band (from 7.944% ± 1.194% to 21.71% ± 3.027%; *n* = 6, *p* = 0.001) of the VTA LFP (Figure 5e). Meanwhile, optogenetic activation of DRN serotonergic terminals in the VTA during REM sleep decreased dopaminergic neuronal firing (from 1.199 ± 0.2613 Spikes/s to 0.5390 ± 0.1550 Spikes/s; *n* = 35, *p* = 0.0028; Figure 5f) and GABAergic neuronal firing (from 23.96 ± 2.371 Spikes/s to 19.33 ± 2.027 Spikes/s; *n* = 32, *p* = 0.0370; Figure 5h), but did not influence glutamatergic neuronal firing (from 0.3500 ± 0.04799 Spikes/s to 0.4604 ± 0.06459 Spikes/s; *n* = 32, *p* = 0.1369; Figure 5j). These results suggest that DRN serotonergic neurons modulating VTA to promote arousal from sleep via dynamically change VTA neuronal firing.

## 4 Discussion

In this study, we found that DRN serotonergic neuronal terminal activity in the VTA was higher in wake than in NREM and REM sleep and changed correlating with sleep–wake switch, consistent with previous studies [6, 20, 21]. Nevertheless, others also reported that serotonergic neurons located in the dorsal part of the rostral and middle DRN firing actively during sleep [22]. Our retrograde tracing results showed that the VTA projecting serotonergic neurons were mainly located in the interfascicular part of the DRN. Moreover, DRN serotonergic neurons have been considered to participate in REM sleep [7, 23, 24], for example, orexin modulated DRN serotonergic neurons contribute to REM sleep cataplexy [23], ablation of central serotonergic neurons decreased REM sleep and attenuated arousal response [24]. However, we found that DRN serotonergic neuronal terminal activity in the VTA was lowest in REM sleep. These studies hint diverse activity of DRN serotonergic neurons during sleep–wake cycle may be based on projection heterogeneous.

The effects of the DRN serotonergic neurons in sleep–wake are still controversial. It has been reported that DRN serotonergic neurons mainly promote wake [1, 2, 3, 4, 5], but also can promote sleep [6, 25]. And, DRN serotonergic neurons projecting to the VTA is a key modulator of balance between reward and aversion [15, 16]. Our previous research found that activating DRN serotonergic neurons reduced the depth of anesthesia, and microinjection of 5‐HT1A or 5‐HT2C receptor into the lateral cerebral ventricle shortened the emergence time from isoflurane anesthesia [18]. In current study, we showed that optogenetic activation of DRN serotonergic terminals in the VTA promoted arousal from sleep and chemogenetic activation of VTA projecting DRN serotonergic neurons increased and stabilized wake. These studies suggest that DRN‐VTA neural pathway plays a wake‐promoting role in sleep–wake process, however, whether it also promotes arousal from general anesthesia needs further investigation.

It has been found that VTA glutamatergic neurons and dopaminergic neurons promote wake from sleep [26, 27, 28], while GABAergic neurons promote sleep [14, 29]. We showed that VTA dopaminergic neuronal firing did not significantly change during the sleep–wake, glutamatergic and GABAergic neuronal firing were higher in wake and REM sleep than in NREM sleep. These results consist with previous study which indicated that the calcium signal activity of VTA glutamatergic and GABAergic were wake and REM active [14], but not completely same with study which indicated calcium signal activity of VTA dopaminergic was highest in REM sleep [28]. Previous studies demonstrated that VTA dopaminergic neurons show high level burst firing in REM sleep and during the consumption of palatable food, but not showed significant firing rate difference [13, 30]. Therefore, the different presentations of ours and other's maybe involved firing patterns changes of VTA dopaminergic neurons.

In this study, we found that optogenetic activation of DRN serotonergic terminals in the VTA mainly increased glutamatergic neuronal firing, decreased dopaminergic neuronal firing, but not changed GABAergic neuronal firing during NREM sleep. Moreover, we also found that optogenetic activation of DRN serotonergic terminals in the VTA decreased dopaminergic and GABAergic neuronal firing, but not changed glutamatergic neuronal firing during REM sleep. Previous studies have found that serotonin has both excitatory and inhibitory effects on dopaminergic neurons [31, 32]. Studies also reported that perfusing serotonin in the VTA inhibited more than 90% of GABAergic neurons [33, 34, 35]. Our results indicated a dynamic change of VTA neuronal firing that influenced by DRN serotonergic neurons in promoting arousal from sleep, however, whether this dynamic change involves VTA microcircuit mechanism still needs to be further clarified. However, we did not transfect NPHR on VTA glutamatergic and GABAergic neurons so that we could use laser stimulation to define them. Using optogenetics to define VTA glutamatergic and GABAergic neurons is more accurate. We defined three types of neuronal firings in the same animal in order to show potential microcircuit dynamics. However, in any case, this is a limitation of this study.

Previous studies showed that 5‐HT1A, 5‐HT1B, 5‐HT2A, 5‐HT2C, 5‐HT3, and 5‐HT4 receptors are expressed in VTA [36]. However, in current study, we did not investigate the receptor mechanisms that mediated the wake‐promoting effect of DRN serotonergic neurons modulate VTA neuronal firing, which need further study.

In all, we found that upregulation of DRN serotonergic afferent in the VTA induces a dynamic change of increase glutamatergic neuronal firing, decreases dopaminergic neuronal firing, and promotes arousal from NREM sleep, meanwhile, a dynamic change of decreases dopaminergic and GABAergic neuronal firing and promote arousal from REM sleep. Our findings provide evidence in understanding the role of DRN serotonergic neurons‐VTA neural pathway in sleep–wake in the view of downstream neuronal firing dynamic changes.

## Author Contributions

Study design: J.L., H.D. Conduct of experiments: Y.W., D.W., Y.H., H.L. Data analysis: Y.W., X.Z., J.L. Data interpretation: Y.W., J.L. Manuscript preparation: Y.W., G.Z., J.L., H.D. Overall project supervision: J.L., H.D.

## Conflicts of Interest

The authors declare no conflicts of interest.

## Supporting information


Figure S1. and S2.

## Data Availability

The data that support the findings of this study are available from the corresponding author upon reasonable request.
