# Supplementary material for: Dorsal Raphe Serotonergic Neurons‐Ventral Tegmental Area Neural Pathway Promotes Wake From Sleep
Source: CNS Neurosci Ther. 2024 Nov 26;30(11):e70141. doi: 10.1111/cns.70141 (PMC11598740; doi:10.1111/cns.70141)
Supplement: Supplementary file 1 — Figure S1. and S2. [file CNS-30-e70141-s001.docx]

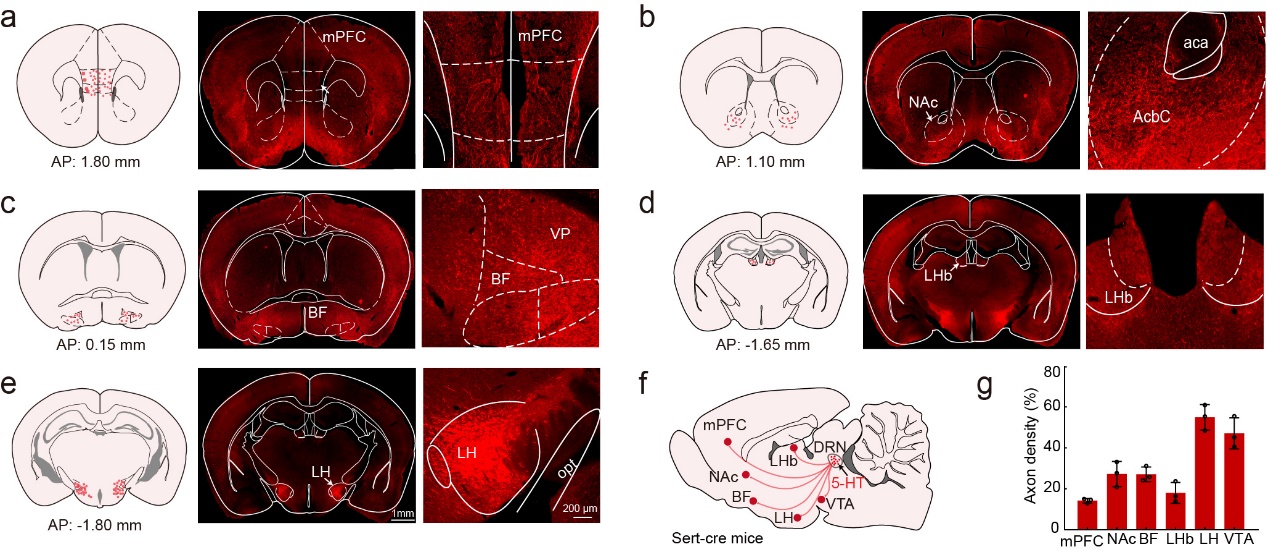


**Figure S1 (related to Figure 1). DRN serotonergic neurons sent projections to many brain regions.** (a) Sample diagram (Left), brain slice image (Middle) and enlarged image from middle (Right) of DRN serotonergic terminal expressing in the mPFC. (b) Sample diagram (Left), brain slice image (Middle) and enlarged image from middle (Right) of DRN serotonergic terminal expressing in the NAc. (c) Sample diagram (Left), brain slice image (Middle) and enlarged image from middle (Right) of DRN serotonergic terminal expressing in the BF. (d) Sample diagram (Left), brain slice image (Middle) and enlarged image from middle (Right) of DRN serotonergic terminal expressing in the LHb. (e) Sample diagram (Left), brain slice image (Middle) and enlarged image from middle (Right) of DRN serotonergic terminal expressing in the LH. (f) Schematic diagram showing DRN serotonergic neurons sent projections to many brain regions. (g) Quantification of DRN serotonergic terminal fluorescence intensity in different brain regions. mPFC, medial prefrontal cortex; NAc, the nucleus accumbens; BF, basal forebrain; LHb, lateral habenula; LHA, lateral hypothalamus; VTA, ventral tegmental area.


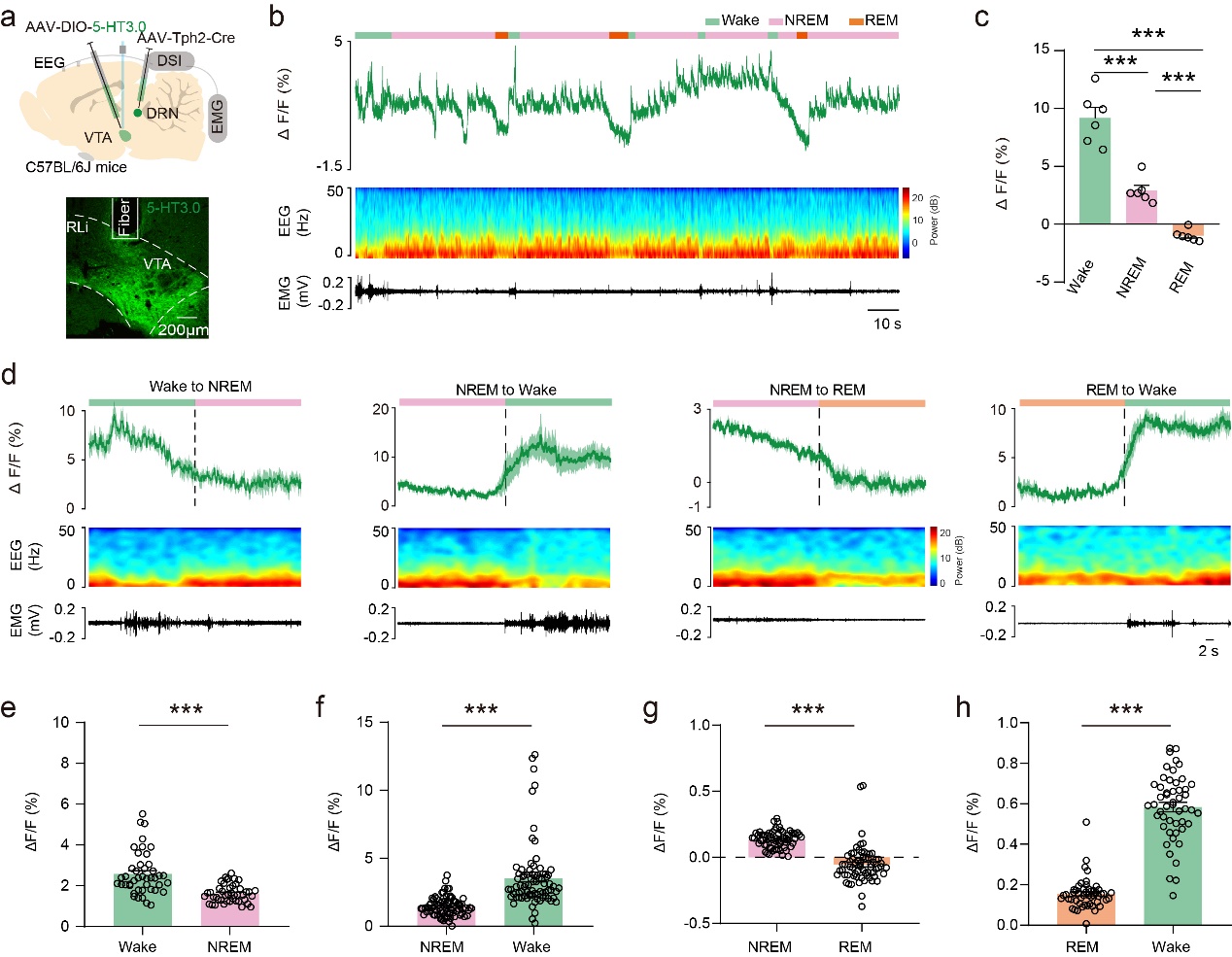


**Figure S2 (related to Figure 1). VTA serotonergic sensor activity changes in the during sleep-wake cycle.** (a) Schematic of virus injection, fiber and DSI implantation and image of 5-HT3.0 expression and optical fiber cannula in the VTA. (b) Representative raw VTA 5-HT3.0 fluorescence intensity trace and relevant EEG power spectra/EMG traces during sleep-wake. (c) Quantification of VTA 5-HT3.0 fluorescence intensity during wake, NREM and REM. (d) Representative raw VTA 5-HT3.0 fluorescence intensity trace and relevant EEG power spectra/EMG traces in states switches during sleep-wake. (e) Quantification of VTA 5-HT3.0 fluorescence intensity in wake switch to NREM sleep. (f) Quantification of VTA 5-HT3.0 fluorescence intensity in NREM sleep switch to wake. (g) Quantification of VTA 5-HT3.0 fluorescence intensity in NREM sleep switch to REM sleep. (h) Quantification of VTA 5-HT3.0 fluorescence intensity in REM sleep switch to wake. ****P*<0.001, VTA, ventral tegmental area; DR, dorsal raphe; EEG, electroencephalogram; EMG, electromyography.
